# Immunomodulatory Effect of Gut Microbiota-Derived Bioactive Peptides on Human Immune System from Healthy Controls and Patients with Inflammatory Bowel Disease

**DOI:** 10.3390/nu11112605

**Published:** 2019-10-31

**Authors:** Samuel Fernández-Tomé, Alicia C. Marin, Lorena Ortega Moreno, Montserrat Baldan-Martin, Irene Mora-Gutiérrez, Aitor Lanas-Gimeno, José Andrés Moreno-Monteagudo, Cecilio Santander, Borja Sánchez, María Chaparro, Javier P. Gisbert, David Bernardo

**Affiliations:** 1 Hospital Universitario de La Princesa, Instituto de Investigación Sanitaria Princesa (IIS-IP), Universidad Autónoma de Madrid, 28006 Madrid, Spain; 2 Centro de Investigación Biomédica en Red de Enfermedades Hepáticas y Digestivas (CIBEREHD), 28029 Madrid, Spain; 3 Instituto de Productos Lácteos de Asturias (IPLA-CSIC), 33300 Asturias, Spain; 4 Mucosal Immunology Lab, Instituto de Biología y Genética Molecular (IBGM, Universidad de Valladolid-CSIC), 47003 Valladolid, Spain

**Keywords:** antigen presenting cells, bioactive peptides, human, IBD, immunomodulation, microbiota

## Abstract

Bioactive peptides secreted by probiotic *Bifidobacterium longum* (peptide B7) and opportunistic pathogen *Bacteroides fragilis* (peptide B12) modulate the intestinal cytokine milieu in health. Here, we characterized their capacity to modulate both the mucosal cytokine production and the phenotype of circulating antigen presenting cells (APCs) in active inflammatory bowel disease (IBD). The IBD mucosa produced higher levels of pro-inflammatory cytokines referred to healthy controls (HCs). Peptides B7 and B12, however, did not ameliorate the mucosal cytokine milieu in IBD. Human circulating APCs (B-cells, monocytes, plasmacytoid dendritic cells (pDCs), and conventional dendritic cells (cDCs)) were characterized by flow cytometry in presence/absence of the peptides. Circulating B-cells, monocytes, and cDCs from IBD patients were more activated than those from HCs. Peptide B7, but not B12, decreased CCR2 expression on all APC subsets from HC, but not IBD patients. Moreover, both peptides tend to further increase their pro-inflammatory profile in IBD. In summary, IBD patients display mucosal and circulating APC pro-inflammatory properties. Peptide B7 immunomodulatory capacity elicited over circulating APCs from HC, but not IBD patients, suggests the presence of disrupted modulatory mechanisms for this peptide in IBD. Future studies should address the effect of bacteria-derived immunomodulatory peptides in non-inflamed (quiescent) IBD patients.

## 1. Introduction

Inflammatory bowel disease (IBD), including Crohn´s disease (CD) and ulcerative colitis (UC), is an immune-mediated disorder that results in chronic inflammation of the gastrointestinal tract. IBD represents a serious health problem, affecting more than 2.2 and 1.6 million inhabitants in Europe and USA, respectively. Indeed, recent evidence suggests that its prevalence in Western countries may be as high as 1/125 [1]. Moreover, IBD incidence is increasing in the newly industrialized countries that have adapted a Westernized lifestyle [2]. Although IBD aetiology remains partly understood, it is influenced by a combination of genetic, immunological, and environmental factors. Indeed, growing evidence supports the role of gut microbiota, diet, intestinal barrier function, and mucosal immune response as key elements in IBD pathogenesis [3,4,5].

Favouring the control of gut homeostasis, several probiotic species of *Lactobacillus* and *Bifidobacterium* have been found to increase the cytotoxic activity of natural killer cells and the phagocytosis of macrophages, as well as mediate the adaptive immune responses elicited by subsets of dendritic cells (DCs), B- and T-cells, and enterocytes [6,7,8,9]. However, beyond their direct contact with mucosal systems, microbiota might also regulate host immunity by soluble chemical mediators [10]. These immune molecular effectors include cell wall components, exopolysaccharides, short-chain fatty acids, conjugated linoleic acid, bacteriocins, extracellular bacterial proteins, and bioactive peptides [11]. In this regard, bioactive peptides derived from food, gut microbiota, or probiotics have already been recognised as immunomodulatory compounds [12,13,14].

The “Mechanism of Action of the Human Microbiome” (MAHMI) database (http://www.mahmi.org) was recently designed as a bioinformatics resource for the screening of potential immunomodulatory and anti-proliferative peptides encrypted in the human gut metaproteome [15]. Indeed, the MAHMI database has already allowed the identification of several bioactive peptides [16,17,18]. Hence, Hidalgo-Cantabrana and colleagues recently identified peptide derived from *Bacteroides fragilis* YCH46 (peptide B12), which polarized Th17 and Th22 responses in human peripheral blood mononuclear cells (PBMCs) from healthy controls (HCs) [16]. Through MAHMI prediction, we previously screened a set of 20 bacterial peptides for their use as non-invasive IBD biomarkers and immunomodulatory compounds, and highlighted the tolerogenic potential of peptide from probiotic *Bifidobacterium longum* subsp. *longum* ATCC 15707 (peptide B7) in the healthy human mucosa [19]. Therefore, we aimed here to investigate the capacity of both peptides (B7 and B12) to modulate the mucosal cytokine production in IBD patients. Moreover, we also studied their effect over the phenotype of circulating antigen presenting cells (APCs) from HC and IBD patients, aiming to identify the specific cellular subset targeted by the peptides.

## 2. Materials and Methods

### 2.1. Patients and Biological Samples

Intestinal biopsies from HC and IBD patients were obtained during colonoscopy. HCs were referred owing to rectal bleeding or colorectal cancer screening. In all cases, they had macroscopically and histologically normal mucosa. In IBD patients, colonoscopy was performed owing to clinical practice for disease diagnosis or monitoring. IBD biopsies included patients with active (inflamed) UC (Mayo endoscopic score > 1) and CD (simplified endoscopic activity score for CD > 3), as defined by the endoscopic assessment. Blood samples were obtained from HCs without autoimmune disease or malignancy as well as from endoscopically active IBD patients. Therefore, samples were obtained from a total of 20 HC (Supplementary Table S1 and Supplementary Table S2) and 17 active IBD patients (Supplementary Table S3 and Supplementary Table S4). In all cases, samples were obtained following written informed consent after ethics approval from the Ethics Committee at La Princesa Hospital (Madrid, Spain) (AM-A_BacPep-2017).

Lipopolysaccharide (LPS)-free peptides (B7 sequence: WIEAVGYSLTQHPDPELEK; B12 sequence: LPLAFFVLTFLWALILR) were chemically synthesized (>95% purity) by Genecust facilities (Ellange, Luxemburg). Freeze-dried peptides were stored at −80 °C until used.

### 2.2. Biopsy Processing and Culture

Intestinal biopsies from HC (*n* = 10; Supplementary Table S1) and active IBD patients (*n* = 8; Supplementary Table S3) were obtained during the colonoscopy. In contrast to inflamed mucosa from IBD patients, the mucosa was not inflamed in any case in the colonic samples from HCs. In all cases, biopsies were immediately preserved in ice-chilled complete medium (Dutch modified RPMI 1640 (Sigma-Aldrich, Dorset, UK) containing 100 μg/mL penicillin/streptomycin, 2 mM L-glutamine, 50 μg/mL gentamicine (Sigma-Aldrich), and 10% foetal calf serum (TCS cellworks, Buckingham, UK)), and processed within 30 min. Biopsies from HC and IBD patients were cultured in resting conditions (one biopsy per 1 mL of complete medium per well in 24-well culture plates) for 18 h at 37 °C. Biopsies from IBD patients were also cultured in parallel in the presence of bacterial peptides B7 or B12 at a concentration previously described to be optimal in our culture system (1 µg/mL) [19]. In all cases, the medium was harvested after culture and centrifuged, and the cell-free culture supernatants were immediately cryopreserved at −80 °C until analysis.

### 2.3. Human Intestinal Cytokine Milieu

Prior to analysis, all HC and IBD biopsy supernatants were thawed once and centrifuged to remove any debris. Cytokine determination was performed using the Human Inflammation Panel (LEGENDplex™, BioLegend, San Diego, CA, USA), following the manufacturer’s specifications. This panel allows the simultaneous quantification of 13 human inflammatory cytokines/chemokines, including IL-1β, IFN-α2, IFN-γ, TNF-α, CCL-2 (chemokine (C–C) motif ligand 2), IL-6, IL-8 (chemokine (C–X–C) motif ligand 8), IL-10, IL-12p70, IL-17A, IL-18, IL-23, and IL-33, based on fluorescence-encoded beads suitable for flow cytometry. Multiplex immunoassay was performed as previously described [20], with standard curves used for all cytokines (2.4–10,000 pg/mL), except IL-33 (12.2–50,000 pg/mL). Samples were acquired on a BD FACSCanto™ II flow cytometer (BD Biosciences) and the generated files were analysed using the Biolegend´s LEGENDplex™ Data Analysis Software (version 8.0). IL-8 was over the detection limit in all of the cases, while IL-12p70 was below the lower threshold in all samples. Both cytokines were thus excluded from the analysis.

### 2.4. Blood Processing and Culture

PBMCs from HC (*n* = 10; Supplementary Table S2) and active IBD patients (*n* = 9; Supplementary Table S4) were obtained following blood centrifugation over Ficoll–Paque PLUS (Amersham Biosciences, Chalfont St. Giles, UK). PBMCs were washed twice in RPMI medium and stained (time 0 h) with fluorochrome-conjugated antibodies, as explained below. PBMCs were also cultured overnight (1 × 10^6^ PBMC in 1 mL of complete medium per tube in polystyrene test tubes (Corning Inc., Corning, NY, USA)) in the absence (resting conditions) and presence of bacterial peptides B7 or B12 (1 µg/mL). Following 18 h culture, PBMCs were washed with PBS containing 1 mM EDTA and 0.02% sodium azide (FACS buffer) and stained with fluorochrome-conjugated antibodies, as detailed below.

### 2.5. Antibody Labelling and Flow Cytometry

PBMCs were stained with monoclonal antibodies and characterized by flow cytometry. Supplementary Table S5 shows the specificity, clone, fluorochrome, and manufacturer of the monoclonal antibodies used in the study. PBMCs were labelled in FACS buffer in ice and in the dark for 20 min. For the assessment of intracellular cytokines (IL-10 and IL-1β), PBMCs were permeabilized (Leucoperm, Abd Secrotec, Oxford, UK) following surface staining and stained with intracellular antibodies. PBMCs were further washed in FACS buffer, fixed with 2% paraformaldehyde in FACS buffer on ice in the dark for 10 min, and washed again in FACS buffer before they were stored at 4 °C prior to acquisition on the flow cytometer.

PBMCs were acquired on a BD LSR-Fortessa™ II flow cytometer (BD Biosciences). All cells were analysed within the singlet viable (>95% viability) fraction. Positive and negative gatings were set by the fluorescence minus one (FMO) method. The results were analysed using FlowJo (version 10.1) (Flowjo LLC, Ashland, USA).

### 2.6. Statistical Analysis

Data were analysed using GraphPad Prism 6.01 software (San Diego, CA, USA) by *t*-test or one-way analysis of variance (ANOVA) (with or without repeated measures) and subsequent Dunnet comparison test, as detailed in the figure legends. *p*-values < 0.05 were considered statistically significant.

## 3. Results

### 3.1. Differential Profile of Mucosal Cytokine Production in HC and IBD Patients

**Figure 1:**
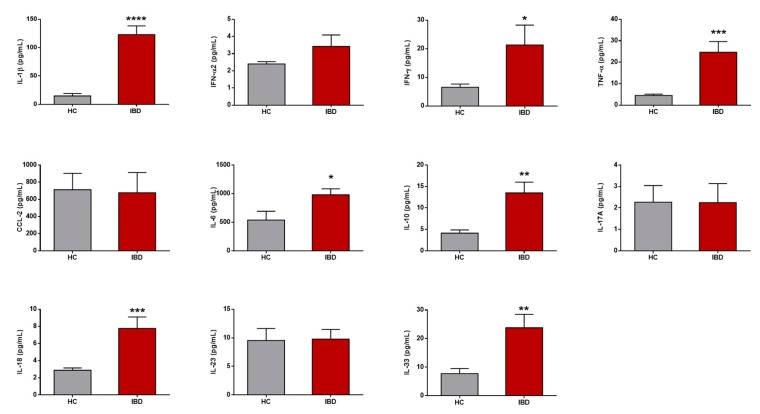
Mucosal cytokines in health and inflammatory bowel disease. Mucosal cytokine levels (pg/mL) in cell-free biopsy culture supernatants from healthy controls (HCs) and patients with inflammatory bowel disease (IBD) following overnight culture in resting conditions. Results are expressed as mean ± SEM. Unpaired *t*-tests were applied for each cytokine. *p*-values < 0.05 were considered significant (* < 0.05, ** < 0.01, *** < 0.001, **** < 0.0001). CCL-2, chemokine (C–C) motif ligand 2. IL, interleukin. IFN, interferon. TNF, tumor necrosis factor.

The intestinal cytokine milieu of HC and active IBD patients was evaluated in biopsy supernatants following overnight culture in resting conditions. IBD patients displayed a mucosal pro-inflammatory profile compared with HCs, with higher levels of IFN-γ and IL-6 (*p* < 0.05), IL-10 and IL-33 (*p* < 0.01), TNF-α and IL-18 (*p* < 0.001), and IL-1β (*p* < 0.0001) (Figure 1). Mucosal levels of IFN-α2, IL-17A, and IL-23 cytokines, together with chemokine CCL-2, were not, however, statistically different between HC and IBD patients.

### 3.2. Bacterial Peptide Conditioning over the IBD Mucosa 

Given that endoscopically active IBD patients display a mucosal pro-inflammatory cytokine milieu (Figure 1), we next determined whether bacterial peptides B7 and B12 may modulate the intestinal cytokine milieu in IBD and restore gut homeostasis as in healthy controls [19]. Hence, cytokine levels were also determined in the IBD culture supernatants following paired overnight culture of the biopsies in the presence of both peptides (Table 1). Secretion of regulatory IL-10 was statistically decreased in IBD patients after conditioning with both peptides (*p* < 0.05). In addition, peptide B7 showed a trend to decrease the secretion of pro-inflammatory IFN-γ (*p* = 0.081) and increase chemoattractant CCL-2 (*p* = 0.076), while peptide B12 displayed a trend to increase the secretion of IL-23 (*p* = 0.125). All other studied cytokines were not affected following culture in any case. Hence, our results suggest that neither peptide B7 nor peptide B12 were capable, in our culture system, of ameliorating the mucosal immune response in active IBD patients.

### 3.3. Characterization of Circulating APC in HC and IBD Patients

Although bioactive peptides B7 and B12 can modulate mucosal immune responses in health [19], they cannot nevertheless restore the cytokine milieu in IBD patients (Table 1). Therefore, we next studied whether these peptides may display immunoregulatory capacity in other culture systems. Hence, we next focused on human APCs as they determine the outcome (pro-inflammatory or tolerogenic) of antigen specific immune responses [21].

**Figure 2:**
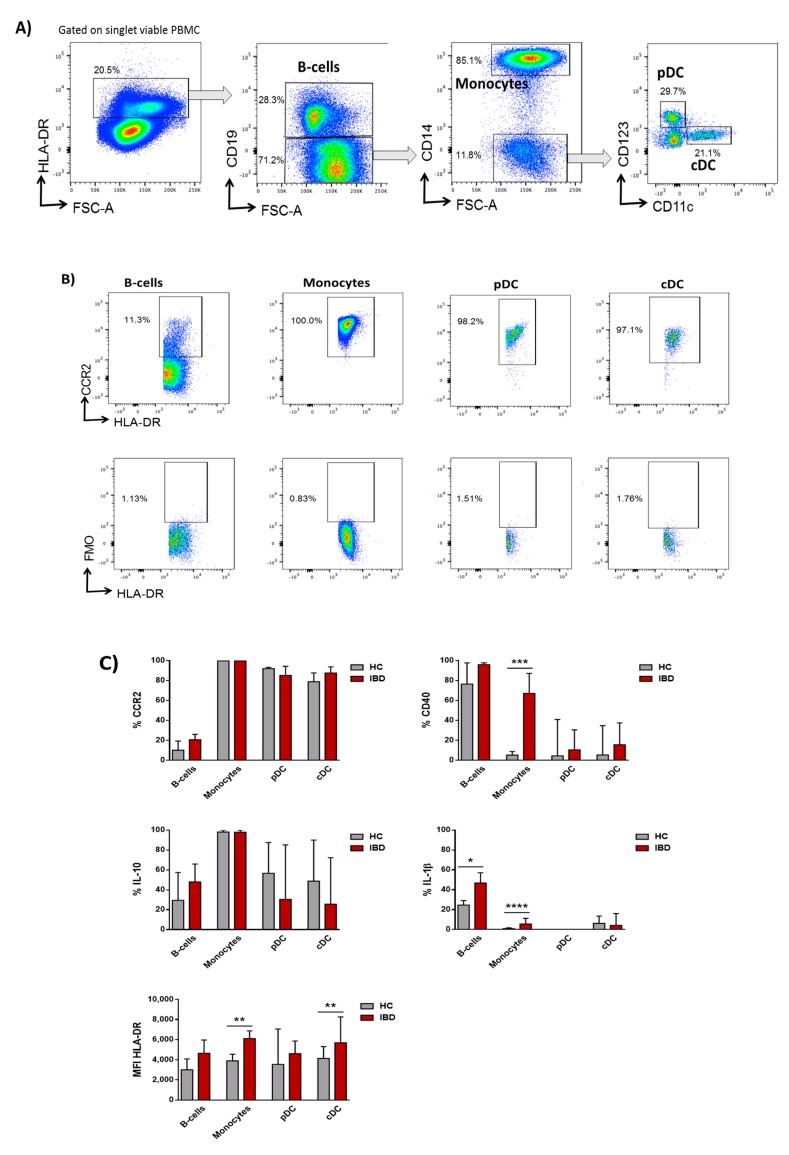
Circulating antigen presenting cells in healthy control and inflammatory bowel disease patients. (**A**) Human antigen presenting cells (APCs) were identified by flow cytometry within singlet viable peripheral blood mononuclear cells (PBMCs) as HLA-DR ^+^. APCs were further divided into B-cells (CD19 ^+^), monocytes (CD19 ^−^, CD14 ^+^), plasmacytoid dendritic cells (pDCs) (CD19 ^−^, CD14^−^, CD123 ^+^, CD11c ^−^), and conventional dendritic cells (cDCs) (CD19 ^−^, CD14 ^−^, CD123 ^−^, CD11c ^+^). (**B**) APC subsets were further characterized for the expression of CCR2, CD40, IL-10, and IL-1β by the fluorescence minus one (FMO) method, as in the example. (**C**) Phenotype of human B-cells, monocytes, pDCs, and cDCs from healthy controls (HCs) and inflammatory bowel disease (IBD) patients based on the basal expression of CCR2, CD40, IL-10, IL-1β, and HLA-DR. Results are expressed as percentage of positive cells (%) for CCR2, CD40, IL-10, and IL-1β or by the median fluorescence intensity (MFI) for HLA-DR in each given subset. The Mann–Whitney test was applied to compare the basal expression of CCR2, CD40, IL-10, IL-1β, and HLA-DR within each subset between HC and IBD patients. *p*-values < 0.05 were considered significant (* < 0.05, ** < 0.01, *** < 0.001, **** < 0.0001).

Human circulating APCs (including B-cells, monocytes, plasmacytoid dendritic cells (pDCs), and conventional dendritic cells (cDCs)) were identified within singlet viable PBMCs by flow cytometry (Figure 2A), and further characterized for the percentage of cells expressing CCR2, CD40, IL-10, and IL-1β by the FMO method (Figure 2B). All cell subsets were also characterized for the expression of HLA-DR. However, given that HLA-DR was used for the gating of the cells (Figure 2A), its expression on each subset was determined by the median fluorescence index (MFI). Although there were no differences in the proportion of circulating APCs between HC and IBD patients (data not shown), they were typically more activated in the latter. Hence, circulating monocytes from IBD displayed higher expression of CD40 (*p* < 0.001) and HLA-DR (*p* < 0.01), and higher production of IL-1β (*p* < 0.0001) (Figure 2C). In a similar manner, circulating cDCs and B-cells expressed higher levels of HLA-DR (*p* < 0.01) and higher production of IL-1β (*p* < 0.05), respectively (Figure 2C).

Moreover, the differences described in the phenotype and cytokine profile of circulating APCs between HC and IBD patients were maintained following the 18 h culture (Supplementary Figure S1). Indeed, overnight culture further activated the expression of CD40 and HLA-DR in cDC and B-cells, respectively, from IBD patients, in agreement with their higher pro-inflammatory phenotype.

### 3.4. Immunomodulatory Effect of Bacterial Peptides over Circulating APC from HC and IBD 

Given that blood APCs from IBD patients display a pro-inflammatory profile, we next evaluated the immunomodulatory potential of peptides B7 and B12 over their phenotype and cytokine production.

**Figure 3:**
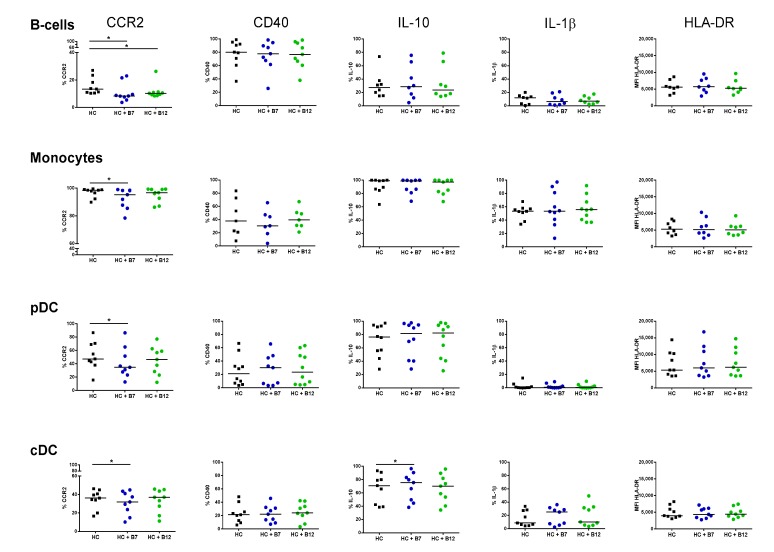
Immunomodulatory effect of bacterial peptides over circulating antigen presenting cells from healthy controls. The effect of bacterial peptides B7 and B12 over the phenotype and cytokine production of B-cells, monocytes, plasmacytoid dendritic cells (pDCs), and conventional dendritic cells (cDCs) from healthy controls (HCs) was determined. Cell subsets were identified as in Figure 2, and characterized following overnight culture in the absence (HC) and presence of bacterial peptides B7 (HC + B7) and B12 (HC + B12). Results are expressed as percentage of positive cells (%) for CCR2, CD40, IL-10, and IL-1β or by the median fluorescence intensity (MFI) for HLA-DR in each given subset. The Wilcoxon paired test was applied to determine statistical differences in the levels of each marker within each subset for both peptides versus resting conditions. *p*-values < 0.05 were considered significant (*).

**Figure 4:**
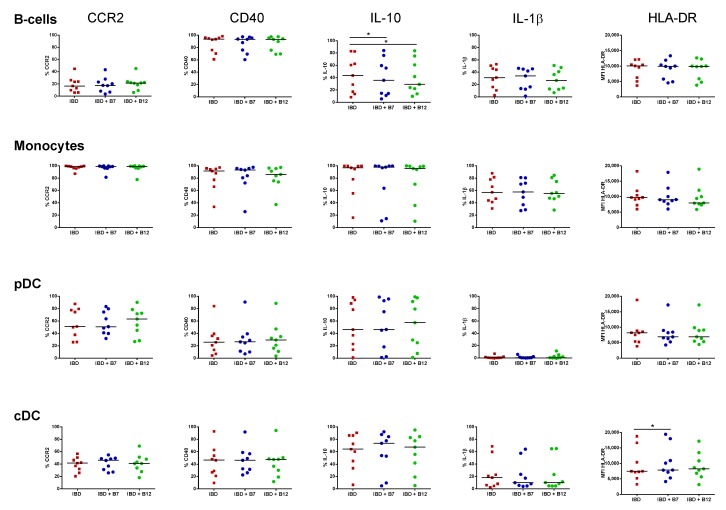
Immunomodulatory effect of bacterial peptides over circulating antigen presenting cells from patients with inflammatory bowel disease. The effect of bacterial peptides B7 and B12 over the phenotype and cytokine production of B-cells, monocytes, plasmacytoid dendritic cells (pDCs), and conventional dendritic cells (cDCs) from patients with inflammatory bowel disease (IBD) was determined. Cell subsets were identified as in Figure 2, and characterized following overnight culture in the absence (IBD) and presence of bacterial peptides B7 (IBD + B7) and B12 (IBD + B12). Results are expressed as percentage of positive cells (%) for CCR2, CD40, IL-10, and IL-1β or by the median fluorescence intensity (MFI) for HLA-DR in each given subset. The Wilcoxon paired test was applied to determine statistical differences in the levels of each marker within each subset for both peptides versus resting conditions. *p*-values < 0.05 were considered significant (*).

Cell subset proportions were not altered following culture in the presence of the peptides (Supplementary Figure S2). Peptide B7 decreased CCR2 expression in all studied subsets (*p* < 0.05), while peptide B12 also lowered its expression on B-cells (Figure 3). This mechanism was, however, restricted to APC from HC as the peptides failed to modulate CCR2 expression in IBD patients (Figure 4). Likewise, both peptides displayed the capacity to modulate the intracellular content of regulatory IL-10, although with different effects between HC and IBD patients. Hence, while peptide B7 increased IL-10 production by cDCs from HCs (*p* < 0.05) (Figure 3), its production was lowered by both peptides on B-cells from IBD patients (*p* < 0.05) (Figure 4). Moreover, in IBD patients, peptide B7 increased the expression of HLA-DR on cDC (*p* < 0.05) and partially expanded IL-1β production of B-cells (*p* = 0.097). Indeed, this pro-inflammatory trend over IL-1β was also elicited by peptide B12 in pDCs from IBD patients (*p* = 0.074) (Figure 4).

## 4. Discussion

Alterations in the intestinal barrier and the commensal microbiota in genetically susceptible individuals is an essential factor in the pathogenesis of IBD. Indeed, the mechanisms mediating the host–microbiota crosstalk, which is disrupted in IBD, remain partially elusive. Hence, it has been suggested that the microbiota modulate gut immunity not only by direct contact with the mucosa, but also through soluble mediators including short-chain fatty acids derived from the metabolism of dietary fiber [22], as well as by peptides encrypted in the intestinal microbial exoproteome [23]. Therefore, we aimed here to study the utility of two gut microbiota-derived bioactive peptides as novel immunomodulatory compounds, which may restore gut homeostasis in patients with IBD.

In this study, we described how active IBD patients display both mucosal and circulating APC pro-inflammatory properties. Our results showed that the inflamed mucosa in these patients produced higher levels of IL-1β, IFN-γ, TNF-α, IL-6, IL-10, IL-18, and IL-33, compared with the non-inflamed cytokine milieu from HC. Hence, these cytokines, among others, have a crucial role in IBD pathogenesis by regulating the initiation, progression, or resolution of the inflammatory process, as well as leading to tissue damage and disease perpetuation in the case of imbalance and deregulatory patterns [24,25].

Interestingly, blood APCs from active IBD patients also reflected a differential profile to HC. Human APCs exert a fundamental function in IBD as they determine the outcome (pro-inflammatory versus tolerogenic) of antigen-specific immune responses [21]. Indeed, mucosal APCs display a pro-inflammatory phenotype and function in IBD, hence driving disease progression [26,27]. Our results revealed that these pro-inflammatory phenotypes can also be observed on their circulating precursors, in agreement with previous observations [28]. Monocytes are the circulating precursors of intestinal macrophages, which mediate gut homeostasis as first line of phagocytic defence and contribute to epithelial renewal, hyporesponsiveness to microbial stimuli, and attenuation of local inflammation [29]. However, this process is disrupted in IBD [30], where we have previously found that there is an increased migration and accumulation of pro-inflammatory CD11c ^high^ monocytes in the mucosa coupled to an abrogated differentiation of these cells into tolerogenic tissue-resident macrophages [27]. In this line, our results showed that activated monocytes from IBD patients had higher expression of CD40 and HLA-DR and produced higher levels of IL-1β, in both fresh samples as well as following overnight culture in resting conditions, also in agreement with previous studies [31]. Similarly, circulating B-cells and cDC subsets also displayed a pro-inflammatory profile in IBD. Microbiota-derived compounds may counteract these immune imbalances and act on IBD by modulating the release of cytokines and chemokines; antibody production; cellular proliferation and activity; signalling pathways; and, ultimately, the immune responses elicited by both the innate and adaptive immune systems [32,33,34].

The protein content of intestinal microorganisms has been recognized as a key molecular player in the dialogue between the host immune system and microbiota [23]. The host–microbiota crosstalk is essential to maintain the mechanisms governing intestinal homeostasis. Nevertheless, these mechanisms are disrupted in IBD patients usually displaying altered microbiota patterns and reduced diversity [35]. Indeed, we previously found that peptides encrypted in the human intestinal microbial-exoproteome may have utility as non-invasive biomarkers for IBD and immunomodulatory compounds in the mucosa from HCs [19]. Hence, although peptides B7 and B12 modulated the cytokine milieu of human lamina propria mononuclear cells in both the presence and absence of pro-inflammatory LPS [19], our results indicate that this is not the case in the inflamed IBD mucosa (Table 1). Indeed, in the present study, both peptides down-regulated the secretion of tolerogenic IL-10 in IBD. However, it has to be highlighted that cellular models used are different, so the peptide effect following culture with lamina propria mononuclear cells may be not reproduced in biopsy culture, which, on the other hand, represents a more physiologically relevant model, as it also includes the intestinal mucus and epithelial layer. Nevertheless, we should also consider the fact that maybe the peptide effect in the mucosa is mild; thus, although they can induce a regulatory profile in the non-inflamed tissue, in the presence of an ongoing inflammation (active IBD patients, as defined in the present study by endoscopic assessment), they cannot ameliorate it. In line with this hypothesis, dietary supplementation with probiotics *Lactobacillus rhamnosus* and *Bifidobacterium breve* led to differential effects in murine colitis models, showing preventive effects when administered prior to the induction of colitis [36], while worsening bloody diarrhoea and inducing expression of TLR2, TLR6, and pro-inflammatory markers in the case in which they are evaluated in the relapse stages of the disease [37]. Similarly, normally harmless strains from *Lactobacillus* have been found to aggravate the undergoing inflammation in human IBD, especially during the acute phase of the disease [38]. Therefore, gut commensals and peptides thereof may be beneficial as novel nutraceutical compounds, which may help to maintain local homeostasis in health or patients in remission, but they might be detrimental as therapeutic agents in active IBD [39,40,41].

Hidalgo-Cantabrana and colleagues previously found that PBMCs from HCs respond to B12 stimulation, increasing the production of IL-6, IL-17A, IL-12p70, IL-22, IL-23, IL-1β, TNF-α, and GM-CSF [16]. In order to complement their finding, we also aimed here to identify by flow cytometry the specific APC subset within PBMCs from HC and IBD patients targeted by peptides B7 and B12. Peptide B7 lowered the expression of CCR2 in all circulating subsets in health. Monocytes have been found to infiltrate the human mucosa in a CCR2-dependent manner [27]. Indeed, cDCs, at least the CD1c ^+^ fraction, are also thought to infiltrate the tissue through this chemokine [42]. Hence, by acting over CCR2, peptide B7 may decrease monocyte and DC migration towards the healthy gut. However, this effect was not mirrored in IBD patients (Figure 4). Given that circulating APCs from IBD patients are more pro-inflammatory that their HC counterparts (Figure 2C), we cannot discard that they are more prone to migrate to the intestinal mucosa to exacerbate the immune response. Therefore, it is possible that intracellular pathways that downregulate CCR2 expression in the presence of the peptides in health may be altered in IBD patients and, consequently, peptides failed to modulate CCR2 in IBD and cells would be more primed to migrate towards the gut.

Peptides slightly modulated the cytokine profile of APCs, although with different effects in HCs and IBD patients. Hence, production of tolerogenic IL-10 within cDCs was increased by peptide B7 in HC. Nonetheless, this property was missing in IBD patients, where both peptides also tend to further active APCs and display a pro-inflammatory trend. Beyond our results with the bioactive peptides B7 from *Bifidobacterium longum* and B12 from *Bacteroides fragilis*, it cannot be discarded that other strains [43] or microbial products from these bacteria may differently modulate the immune response. As an example, the extracellular polysaccharide A from *Bacteroides fragilis* mediates the conversion of CD4 ^+^ T-cells into IL-10-producing Foxp3 ^+^ T-regulatory cells, and thereby provides mucosal tolerance during commensal colonization [44]. However, a disrupted expression of this immunomodulatory microbial compound has been recently identified in IBD patients [45].

## 5. Conclusions

In summary, in the present study, we described how bioactive peptides B7 and B12 from gut commensals were not able, in our culture model, to restore the altered mucosal cytokine profile of patients with active IBD. Indeed, the immunomodulatory capacity of bacterial peptides elicited over circulating APCs from HCs is disrupted in active IBD. Altogether, it is suggested that differential immune mechanisms between healthy controls and IBD patients may abrogate the immunomodulatory tolerogenic potential of bioactive peptides from microbiota in this disease during the active phase. Indeed, given the properties of the studied bacteria peptides, and although they may not be useful to treat inflamed IBD patients, we cannot discard the possibility that they would be beneficial in patients with quiescent disease in order to prevent inflammation flares. In this regard, additional studies about the use of bacterial peptides over the non-inflamed tissue of active IBD patients or in quiescent patients in remission would be needed to further elucidate the immunomodulatory potential of bioactive peptides from microbiota in the context of IBD.

## Figures and Tables

**Table 1:** Peptide effect over the intestinal cytokine milieu.

Cytokines	IBD		IBD + B7		IBD + B12	
	Mean	SEM	Mean	SEM	Mean	SEM
IL-1β	123.0	43.4	120.3	43.4	142.8	41.2
IFN-α2	3.4	0.7	3.4	0.6	3.2	0.6
IFN-γ	21.3	6.9	13.0	5.5	29.9	12.7
TNF-α	24.7	4.9	22.0	7.3	22.2	7.9
CCL-2	677.3	235.6	1032.0	212.4	726.3	215.4
IL-6	980.2	104.4	891.2	141.5	979.2	127.8
IL-10	13.5	2.5	10.0	3.5 *	9.4	2.4 *
IL-17A	2.2	0.9	3.3	2.1	4.8	1.8
IL-18	7.8	1.3	8.6	1.5	6.7	1.5
IL-23	9.8	1.7	19.3	6.6	25.9	8.5
IL-33	23.8	4.7	22.7	5.7	22.0	6.7

Intestinal cytokine milieu of biopsy culture supernatants from inflammatory bowel disease patients in the absence (IBD) and presence of bacterial peptides B7 (IBD + B7) and B12 (IBD + B12). Results are expressed as cytokine levels (pg/mL, mean ± SEM). Paired *t-test* were applied to determine statistical differences in the levels of each cytokine for both peptides *versus* resting conditions. *p*-values < 0.05 were considered significant (*). CCL-2, chemokine (C–C) motif ligand 2. IL, interleukin. IFN, interferon. TNF, tumor necrosis factor.

## Supplementary Materials

The following are available online at https://www.mdpi.com/2072-6643/11/11/2605/s1, Figure S1: Culture effect over circulating antigen presenting cells, Figure S2: Proportion of human circulating antigen presenting cells, Table S1: Demographics of healthy controls for experiments on intestinal cytokine production, Table S2: Demographics of healthy controls for experiments on circulating antigen presenting cells, Table S3: Patient demographics for experiments on intestinal cytokine production, Table S4: Patient demographics for experiments on circulating antigen presenting cells, Table S5: Flow cytometry antibodies.
