# Supplementary material for: Immunomodulatory Effect of Gut Microbiota-Derived Bioactive Peptides on Human Immune System from Healthy Controls and Patients with Inflammatory Bowel Disease
Source: Nutrients. 2019 Oct 31;11(11):2605. doi: 10.3390/nu11112605 (PMC6893616; doi:10.3390/nu11112605)
Supplement: Supplementary file 1 [file nutrients-11-02605-s001.pdf]

Table S1. Demographics of healthy controls for experiments on intestinal cytokine production.

Patient	Age	Gender	Mucosal status	Treatment
HC1	73	Male	Healthy	Untreated
HC2	68	Female	Healthy	Untreated
HC3	72	Female	Healthy	Untreated
HC4	72	Male	Healthy	Untreated
HC5	40	Female	Healthy	Untreated
HC6	43	Male	Healthy	Untreated
HC7	31	Female	Healthy	Untreated
HC8	66	Female	Healthy	Untreated
HC9	42	Female	Healthy	Untreated
HC10	53	Female	Healthy	Untreated

Demographics of healthy controls (HC) used to evaluate the intestinal cytokine production (Figure 1 and Table 1), including age, gender, mucosal status and treatment.

Table S2. Demographics of healthy controls for experiments on circulating antigen presenting cells.

Patient	Age	Gender	Mucosal status	Treatment
HC11	35	Male	Healthy	Untreated
HC12	41	Female	Healthy	Untreated
HC13	36	Male	Healthy	Untreated
HC14	20	Male	Healthy	Untreated
HC15	49	Male	Healthy	Untreated
HC16	50	Female	Healthy	Untreated
HC17	28	Female	Healthy	Untreated
HC18	66	Male	Healthy	Untreated
HC19	30	Male	Healthy	Untreated
HC20	35	Male	Healthy	Untreated

Demographics of healthy controls (HC) used to characterize the circulating antigen presenting cell subsets (Figures 2-4 and Supplementary Figures S1-S2), including age, gender, mucosal status and treatment.

Table S3. Patient demographics for experiments on intestinal cytokine production.

Patient	Age	Gender	IBD type	Endoscopic score[a]		Sample location	Montreal classification[b]		Treatment	Smoking
				CD	UC		CD	UC		
IBD1	25	Male	CD	9	-	Terminal ileum	A2, L1, B1	-	Ustekinumab	Yes
IBD2	58	Male	CD	9	-	Left colon	A3, L2, B1	-	Inflectra	No
IBD3	22	Male	CD	18	-	Right colon	A1, L3, B1	-	Vedolizumab	No
IBD4	32	Male	CD	10	-	Terminal ileum	A2, L3, B3	-	Azathioprine + adalimumab	Yes
IBD5	35	Male	CD	6	-	Left colon	A2, L2, B1	-	Mesalazine	No
IBD6	66	Female	UC	-	2	Left colon	-	E3	Adalimumab	No
IBD7	38	Male	UC	-	3	Left colon	-	E2	Mesalazine	No
IBD8	39	Male	UC	-	3	Left colon	-	E3	Azathioprine + golimumab	No

Demographics of inflammatory bowel disease (IBD) patients used to evaluate the intestinal cytokine production (Figure 1 and Table 1), including age, gender, IBD type, endoscopic score, sample location, Montreal classification, treatment and smoking habit at the time of the colonoscopy. CD: Crohn´s disease; UC: Ulcerative colitis. [a] Mucosal status as defined by endoscopic assessment (CD: simplified endoscopic activity score for CD; UC: Mayo endoscopic score). [b] Montreal classification of IBD [CD, Age at diagnosis: A1 (< 16 years), A2 (17-40 years), A3 (> 40 years); Location: L1 (ileal), L2 (colonic), L3 (ileocolonic); Behaviour: B1 (inflammatory), B2 (structuring), B3 (penetrating). UC, Extent: E1 (proctitis), E2 (left sided), E3 (extensive)].

Table S4. Patient demographics for experiments on circulating antigen presenting cells.

Patient	Age	Gender	IBD type	Endoscopic score[a]		Montreal classification[b]		Treatment	Smoking
				CD	UC	CD	UC		
IBD9	22	Male	CD	18	-	A1, L3, B1	-	Vedolizumab	No
IBD10	32	Male	CD	10	-	A2, L3, B3	-	Azathioprine + adalimumab	Yes
IBD11	25	Male	CD	6	-	A2, L1, B1	-	Azathioprine	No
IBD12	51	Female	CD	6	-	A2, L2, B3	-	Azathioprine + certolizumab pegol	No
IBD13	70	Female	CD	4	-	A3, L3, B1	-	Mesalazine	No
IBD14	46	Male	UC	-	3	-	E2	Mesalazine + inflectra	No
IBD15	47	Male	UC	-	2	-	E2	Mesalazine + prednisolone + etrolizumab	No
IBD16	34	Male	UC	-	2	-	E2	Mesalazine	No
IBD17	40	Male	UC	-	3	-	E3	Mesalazine + azathioprine + etrolizumab	No

Demographics of inflammatory bowel disease (IBD) patients used to characterize the circulating antigen presenting cell subsets (Figures 2-4 and Supplementary Figures S1-S2), including age, gender, IBD type, endoscopic score, Montreal classification, treatment and smoking habit at the time of the colonoscopy.CD: Crohn´s

disease; UC: Ulcerative colitis. [a] Mucosal status as defined by endoscopic assessment (CD: simplified endoscopic activity score for CD; UC: Mayo endoscopic score). [b] Montreal classification of IBD [CD, Age at diagnosis: A1 (< 16 years), A2 (17-40 years), A3 (> 40 years); Location: L1 (ileal), L2 (colonic), L3 (ileocolonic); Behaviour: B1 (inflammatory), B2 (structuring), B3 (penetrating). UC, Extent: E1 (proctitis), E2 (left sided), E3 (extensive)].

Table S5. Flow cytometry antibodies.

Antibody Specificity	Clone	Fluorochrome	Manufacturer
IL-10	JES3-9D7	BV421	Biolegend
HLA-DR	L243	BV570	Biolegend
CCR2	K036C2	BV605	Biolegend
CD40	5C3	BV711	Biolegend
CD123	7G3	FITC	Becton Dickinson
CD14	MoP9	PECF594	Becton Dickinson
CD19	HIB19	PE-Cy5	Becton Dickinson
IL-1β	JK1B-1	APC	Biolegend
CD11c	Bu15	Alexa700	Biolegend

Specificity, clone, fluorochrome and manufacturer of the different monoclonal antibodies used in the study.

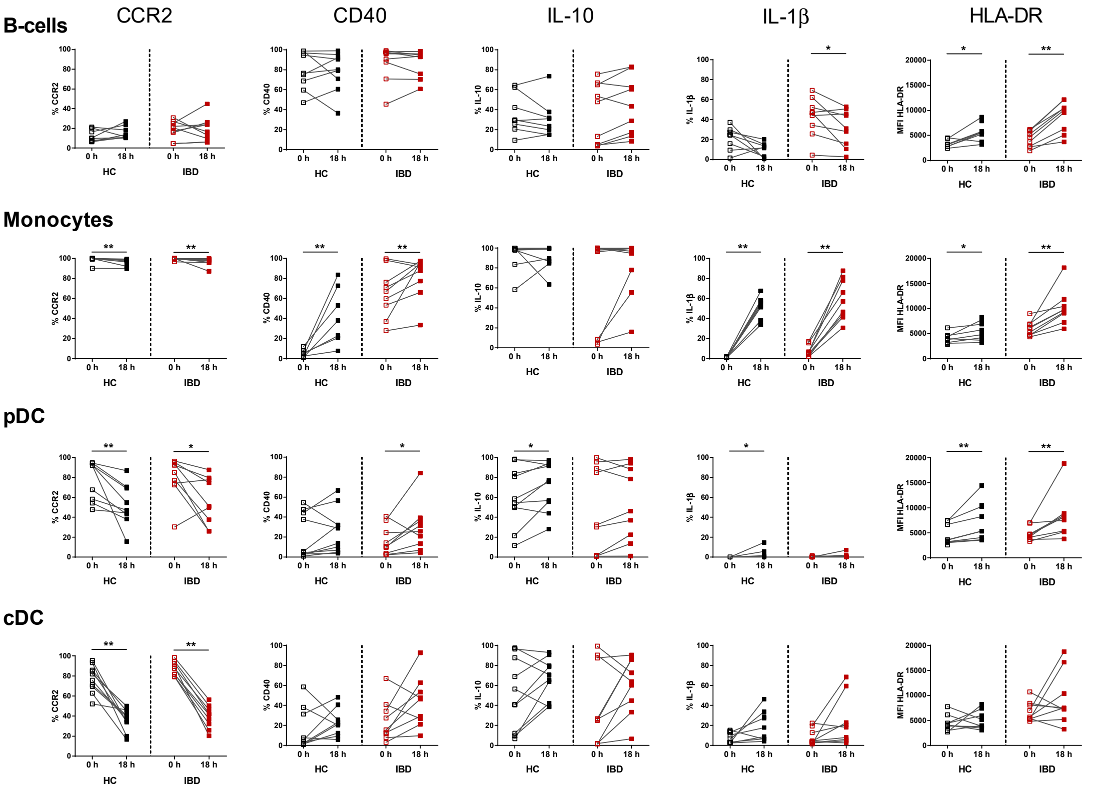

Figure S1. Culture effect over circulating antigen presenting cells. B-cells, monocytes, plasmacytoid dendritic cells –pDC– and conventional dendritic cells –cDC–, from healthy controls (HC) and patients with inflammatory bowel disease (IBD) were identified as in Figure 2. Cells were characterized for the expression of CCR2, CD40, IL-10, IL-1β and HLA-DR within each subset in HC and IBD patients, both in fresh samples (0 h) as well as after overnight culture in resting conditions (18 h). Results are expressed as percentage of positive cells (%) for CCR2, CD40, IL-10 and IL-1β or by the median fluorescence intensity (MFI) for HLA-DR in each given subset. Wilcoxon paired test were applied to compare the basal (0 h) and post-culture (18 h) expression of CCR2, CD40, IL-10, IL-1β and HLA-DR within each subset in HC and IBD patients. P-values < 0.05 were considered significant (* < 0.05, ** < 0.01).

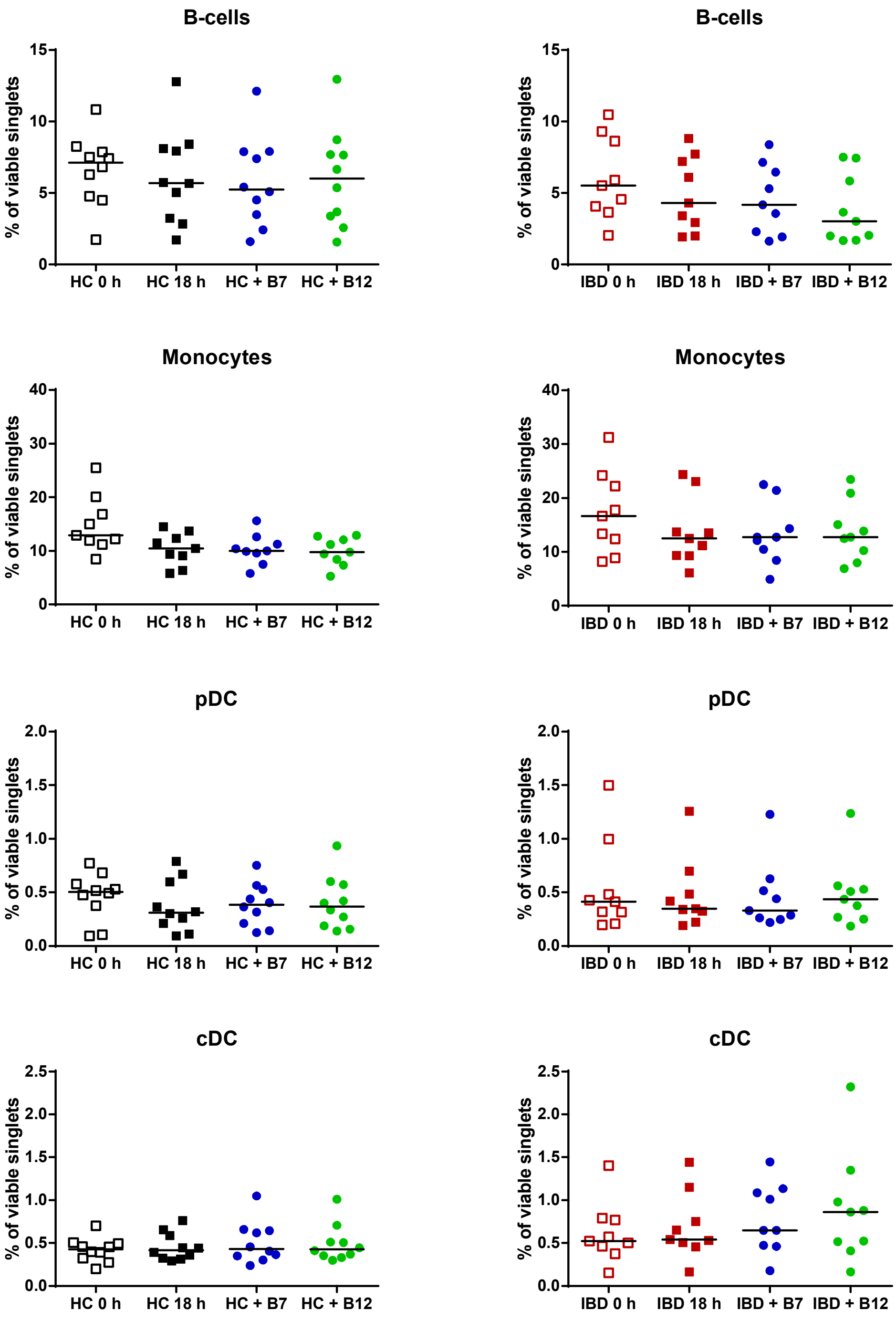

Figure S2. Proportion of human circulating antigen presenting cells. The proportion of B-cells, monocytes, plasmacytoid dendritic cells –pDC– and conventional dendritic cells –cDC– subsets was determined within total singlet viable cells from both healthy controls (HC) and inflammatory bowel disease (IBD) patients. Cell proportion was determined both in fresh samples (0 h), as well as after overnight culture in resting conditions (18 h) or in the presence of bacterial peptides (B7 and B12). Data were analysed statistically by one-way ANOVA with Dunnet comparison test. .
